# Plasma-Induced Heating Effects on Platinum Nanoparticle Size During Sputter Deposition Synthesis in Polymer and Ionic Liquid Substrates

**DOI:** 10.1021/acs.langmuir.1c01190

**Published:** 2021-07-13

**Authors:** Rosemary Brown, Björn Lönn, Robin Pfeiffer, Henrik Frederiksen, Björn Wickman

**Affiliations:** † Chemical Physics, Department of Physics, Chalmers University of Technology, Gothenburg 412 96, Sweden; ‡ MC2, Department of Microtechnology and Nanoscience, Chalmers University of Technology, Gothenburg 412 96, Sweden

## Abstract

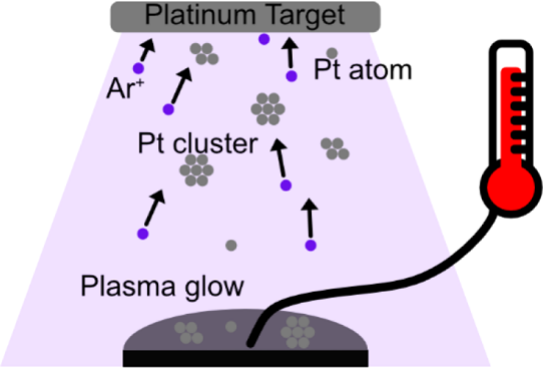

Nanoparticle catalyst materials are becoming ever more important in a sustainable future. Specifically, platinum (Pt) nanoparticles have relevance in catalysis, in particular, fuel cell technologies. Sputter deposition into liquid substrates has been shown to produce nanoparticles without the presence of air and other contaminants and the need for precursors. Here, we produce Pt nanoparticles in three imidazolium-based ionic liquids and PEG 600. All Pt nanoparticles are crystalline and around 2 nm in diameter. We show that while temperature has an effect on particle size for Pt, it is not as great as for other materials. Sputtering power, time, and postheat treatment all show slight influence on the particle size, indicating the importance of temperature during sputtering. The temperature of the liquid substrate is measured and reaches over 150 °C during deposition which is found to increase the particle size by less than 20%, which is small compared to the effect of temperature on Au nanoparticles presented in the literature. High temperatures during Pt sputtering are beneficial for increasing Pt nanoparticle size beyond 2 nm. Better temperature control would allow for more control over the particle size in the future.

## Introduction

Platinum (Pt) nanoparticles have high catalytic relevance and can be produced using a variety of methods from chemical synthesis to more physical methods. Due to the ever-growing importance of Pt nanoparticles, alternative techniques that avoid the use of harsh chemical precursors are being developed. The method of sputter deposition into liquids in vacuo is of particular importance because it removes oxygen and other contaminants which can alter nanoparticle catalytic activity.

The technique of sputter deposition into liquids was first performed by Ye^1^ in 1996 after which it has been growing in interest. So far, many different materials have been produced such as Au,^2−10^ Pt,^11−18^ Pd,^12^ Ag,^19^ and various alloy and core shell configurations.^20−24^ In order to modify and change the size of the nanoparticles, there are various methods and techniques presented in the literature, and the substrate temperature seems to be the most effective with Au nanoparticles.^4,5,7^ There has been some indication that the liquid substrate temperature affects Pt nanoparticle size much less than for Au and Cu,^20^ however it is not known to what extent. As these nanoparticles are extremely useful catalysts, it would be helpful to tailor their size for the relevant application.^11^

Pure Pt nanoparticles have been successfully produced in high- and low-weight poly(ethylene glycol) (PEG)^11,12,14,15^ and the ionic liquids TPMA TFSI^14^ and MePrN Tf2N.^13,16^ The resultant nanoparticles have all been small, around 2 nm in diameter, and there has been some investigation into the effect of sputtering parameters on their size. Deng^11^ found that larger Pt nanoparticles formed with higher sputtering currents in PEG 600. There was additional growth postsputtering after a week or two; however, the Pt nanoparticles were stable for months, indicating that PEG is a good stabilizer and liquid substrate for the production of Pt nanoparticles. The same group has also sputtered Au and PtAu alloys^21^ and found that pure Au nanoparticles agglomerate in PEG, so metal–liquid interactions inside the liquid or on the surface are important for stability.

It has been suggested that the cation alkyl chain length of ionic liquids, in addition to influencing the steric stabilization, can also have an impact on the particle size of metal nanoparticles.^25^ Hatakeyama^6^ studied the alkyl chain length influence on Au nanoparticle size, for a set of 1-alkyl-3-methylimidazolium tetrafloroborate (with alkyl chain lengths of 2, 4, and 8) ionic liquids and found Au nanoparticle size to decrease with increased alkyl chain length. The same group later repeated the study for a range of temperatures, now also including another anion (triflate).^7^ They found the alkyl chain length effect on Au nanoparticle size to be temperature-dependent. At low substrate temperatures, the effect was nearly indistinguishable for varying chain lengths but became more significant for higher temperatures.

Temperature effects can overshadow liquid substrate composition trends, although this has mostly been investigated for Au nanoparticles. Hatakeyama showed clearly that higher liquid temperatures significantly increased Au nanoparticle size in PEG^4^ and in ionic liquids.^3−5,7,8^ Increasing PEG 600 from room temperature to 60 °C increased the size of Au nanoparticles by nearly 2.5 times^4^ and increasing to 50 °C in Emim Tf produced nanoparticles two times larger.^7^ Thus, there is general consensus that higher temperature results in larger particle size of Au nanoparticles.^26^

The effect of temperature on Pt nanoparticle size in liquid substrates however has not been studied in detail. Deng and coworkers^11,20^ found a correlation between current and Pt nanoparticle size in PEG 600 which indicates a temperature dependence on size. They also mentioned that they controlled the temperature, but this is actually tricky to do in a vacuum chamber when plasma is present. It is probable that higher currents could lead to higher liquid temperatures in PEG 600, just as Hatakeyama^4^ found with Au nanoparticles. So far, there has been little study linking temperature to the size of Pt nanoparticles sputtered into other liquid substrates, such as ionic liquids. Cha et al.^15^ sputtered Pt into pyrrolidinium-based ionic liquids and found that there were no Pt particles present without annealing at 200 °C after sputtering. This shows that heat is an important factor also in Pt nanoparticle production.

There has been little discussion and description on the magnetron and plasma configuration inside the sputter chamber for the published work thus far. Plasma can heat its surroundings, and even with a water-cooling system, the temperature of any substrate or substrate surface can be hard to control due to bombardment by ions, atoms, and electrons. In this work, we use an unbalanced magnetron that heats the liquid substrate during operation, and we monitor the temperature inside the liquid during sputtering. A temperature increase of over 150 °C can be achieved, and its effect on particle size is presented. This manuscript shows that Pt nanoparticles can be produced in imidazolium-based ionic liquids and PEG and that although temperature has an effect on the particle size, the effect is much less pronounced than for other metal nanoparticles, such as Au, presented previously.

## Experimental Methods

### Liquid Substrates

Three different ionic liquids, 1-ethyl-3-methylimidazolium triflate (Emim Tf), 1-decyl-3-methylimidazolium triflate (Dmim Tf), and 1-decyl-3-methylimidazolium bis(trifluoromethylsulfonyl)imide (Dmim Tf2N), and the polymer polyethylene glycol 600 (PEG 600) were used as liquid substrates in the present study. All ionic liquids were more than 99% pure and purchased from Iolitec GmbH, and PEG 600 was purchased from Alfa Aesar and 95% pure. Ionic liquids representing a range of alkyl chain lengths (Emim Tf and Dmim Tf) were chosen to elucidate any Pt nanoparticle size dependence on the alkyl chain length. Dmim Tf2N was chosen to compare two long-chained ionic liquids with different anions.

### Sputter Deposition into Liquid Substrates

A custom-built sputter coater was used to sputter Pt into liquid substrates, the base pressure of the system was around 1 × 10^–7^ to 9 × 10^–7^ mbar, and the Argon pressure varied between 0.6 and 0.7 Pa with a flow rate of 30 sccm. Sputtering was performed on 200 μL of the liquid substrate dropped onto a 1-inch glass wafer and rested on a stainless-steel Petri dish at 8 cm working distance from the target material. All samples were pumped for 12 h or longer to remove water. Deposition was performed in runs of 3 × 300 s with 20 min in between each run, and resting was to allow for cooling. For the most part, a power of 50 W (for which current and voltage varied: *I* = 114–119 mA and *U* = 424–437 V due to slight pressure changes) was used. 20 W (*I* = 51 mA and *U* = 373 V) and 65 W (*I* = 145 mA and *U* = 444 V) were used to test the effect of power on Pt nanoparticles in PEG. Prior to each deposition, each wafer was sonic cleaned in acetone, ultrapure water, and IPA (VWR, >99.5%). For temperature measurements, a K-type shielded thermocouple was inserted into the liquid during the entire sputtering duration, including rest periods. A schematic of this can be found in Supporting Information.

### Transmission Electron Microscopy

Holey carbon grids were used to image the nanoparticles in an FEI Tecnai T20 microscope at 200 kV and a high-resolution FEI Titan 80–300 microscope at 300 kV. Grids were prepared by dropping 5–10 μL of the sample on the grid surface which was left for 4 h for particles to attach to the holey carbon support. After this, the grid was washed dropwise with acetonitrile for 45 min, around 5 drops every 3 min.

Image J was used to process the images, and a combination of the particle sizer plugin (Thorsten Wagner) and manual sizing was used to size over 175 particles per sample. Small particle sizes and high noise at high magnifications meant that smaller particles were difficult to size, and this systematic error would be the same for each sample, regardless of this, our transmission electron microscopy (TEM) results give a good indication to the actual nanoparticle size. Size measurement results were fit with a log-normal distribution to treat all the data the same.

### Thermogravimetric Gas Analysis

Thermogravimetric gas analysis (TGA) (TGA/DSC3+ combined, Mettler Toledo) was performed on the liquid substrates and Pt nanoparticle samples. For TGA, liquid substrates were pipetted into 100 μL Al crucibles. Sample mass was then measured in the temperature range 30–200 °C with respect to a blank crucible.

### Small-Angle X-ray Scattering

A Mat/Nordic instrument from SAXSLAB was used to obtain small-angle X-ray scattering (SAXS) data of the suspended Pt nanoparticles. Monochromatic X-rays of 0.154 nm wavelength were produced by a Rigaku 003+ Cu-radiation source. The detector was a Pilatus 300K. Samples were prepared in 1 mm glass capillaries that were subsequently mounted in an ambient capillary plate sample holder. Before the preparation in capillaries, the 200 μL samples were diluted by another 500 μL of the respective substrate liquid to increase transmission. During measurements, the sample to detector distance was within the range of 125.8–127.5 mm.

Data analysis was performed in Sasview 5.0.3. Prior to fitting, corrections for the background and the difference in absorbance of the nanoparticles and the liquid were made. The resulting intensities of scattering from the Pt nanoparticles (*I*_Pt_) in the different liquids were attained by
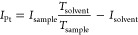
1where *I*_sample_ and *I*_solvent_ are the intensities observed for the Pt-containing liquid samples and their corresponding pure liquids, respectively, while *T*_solvent_ and *T*_sample_ are the transmissions measured for the pure liquids and Pt-liquid samples, respectively.

Fits were obtained by fitting a built-in spherical model based on scattering theory introduced by Guinier^27^ to the data. In the case of Pt in Dmim Tf and Dmim Tf2N samples, a Hayter–Penfold rescaled mean spherical approximation structure factor^28,29^ was added alongside the form factor.

### Post Sputtering Heat Treatment

To examine if liquid temperature induces nanoparticle growth after sputtering, Pt nanoparticles in PEG and Emim Tf were subjected to heating in a silicon oil bath. In this case, sputtering was performed for 3 × 300 s at 50 W in both liquid substrates. The resultant solutions were collected in glass vials and immersed in silicone oil which was heated from room temperature to 165 °C and maintained at that temperature for 1 h.

## Results and Discussion

**Figure 1:**
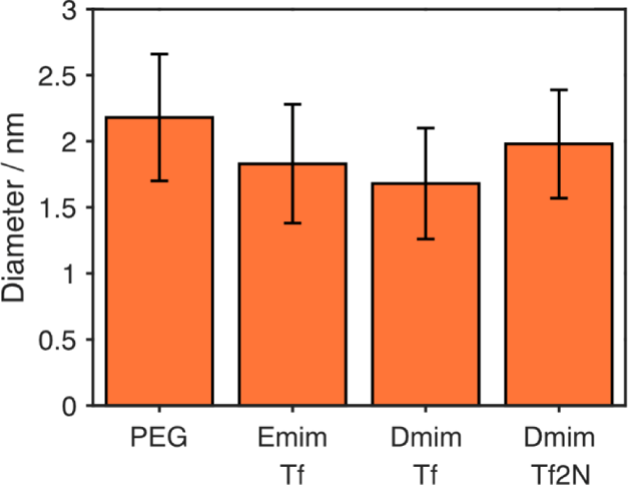
Mean size of Pt nanoparticles produced in PEG, Emim Tf, Dmim Tf, and Dmim Tf2N, seen from analysis of TEM images. The sizes are all within the standard deviation of each other.

**Figure 2:**
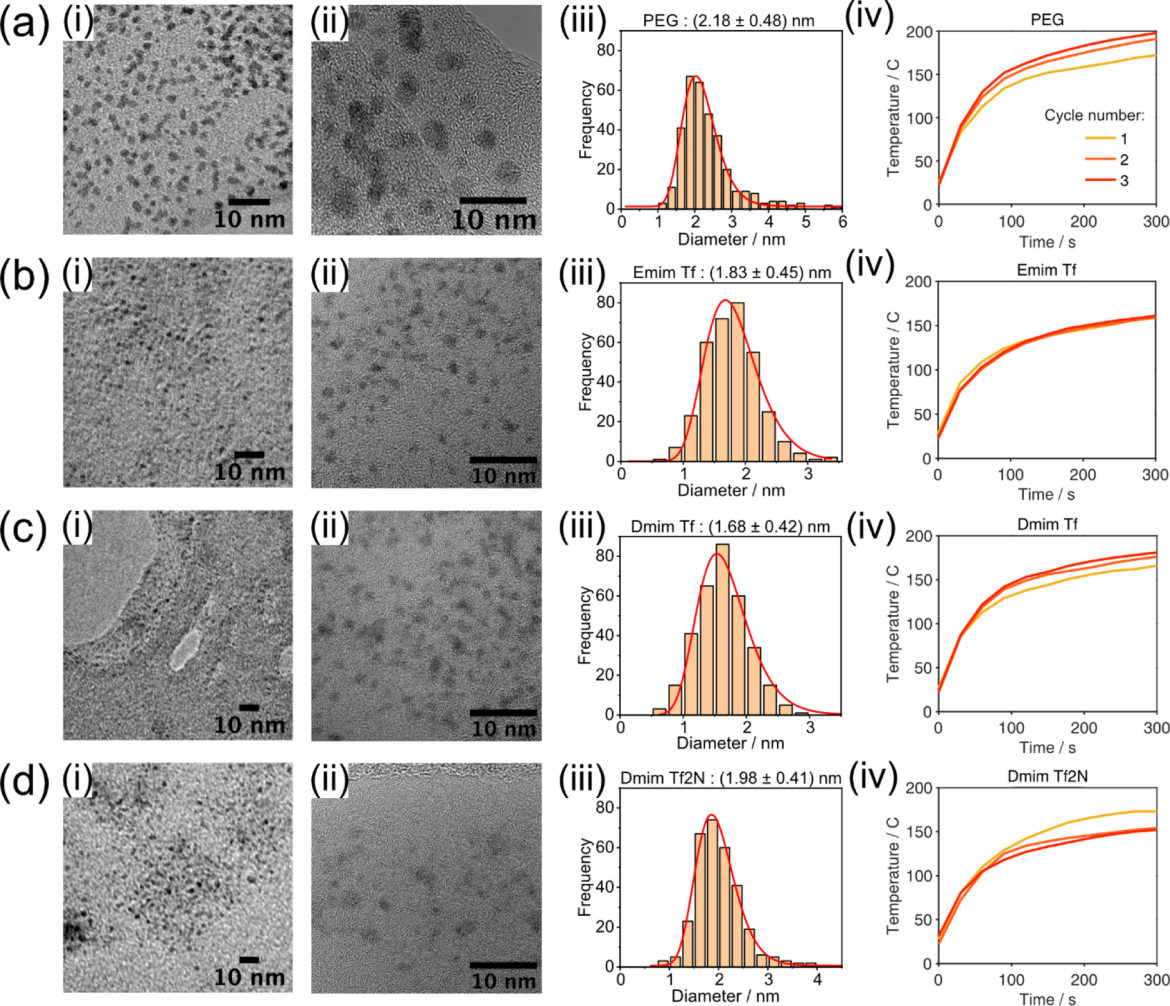
TEM images (i), high-resolution TEM (ii), resultant size distribution (iii), and substrate temperatures during sputtering (iv) of Pt nanoparticles produced in (a) PEG 600, (b) Emim Tf, (c) Dmim Tf, and (d) Dmim Tf2N. Particles were sized using a combination of Image J particle sizer and individual measurement by hand, and data were fit with a log-normal distribution.

Pt nanoparticles were produced successfully in all liquids, and their mean sizes are shown in Figure 1. All particles produced were around 2 nm in diameter, differing only by less than 0.5 nm between liquid substrates with the size in PEG 600 > Dmim Tf2N > Emim Tf > Dmim Tf. All sizes were within the standard deviation of each other showing that there is not much difference between the particle formation in these liquid substrates. TEM was used to obtain the mean nanoparticle size and to also observe their crystallinity. Figure 2 shows typical standard (i) and high-resolution (ii) TEM images for each liquid. These were used to size the Pt nanoparticles. The resultant size distributions are shown in Figure 2iii.

**Figure 3:**
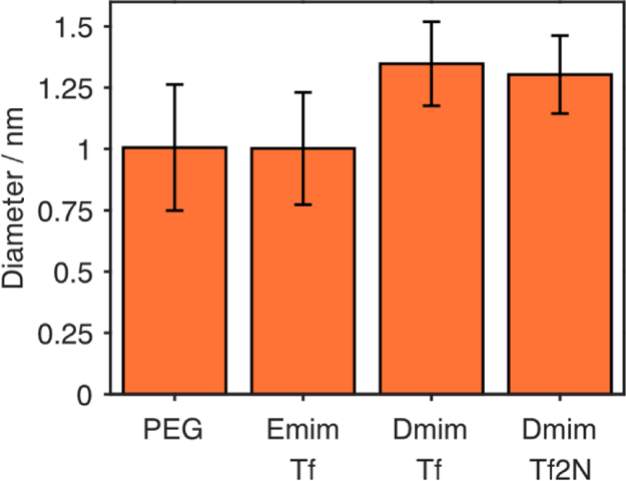
Mean size of Pt nanoparticles produced in PEG, Emim Tf, Dmim Tf, and Dmim Tf2N, obtained from SAXS fits. The sizes are all within the standard deviation of each other.

Pt nanoparticles in PEG (Figure 2a) were the easiest to focus on in the TEM showing how successful the grid rinsing protocol was. There seemed to be the fewest Pt nanoparticles in Dmim Tf2N (Figure 2d), which is likely due to the difficulty to remove it completely from the nanoparticle surface and grid itself; however, it could also be that a larger fraction of nanoparticles were too small to observe. Emim Tf and Dmim Tf (Figure 2b,c) both showed many particles but with unclear edges. This highlights the difficulty in analyzing such small nanoparticles with TEM, a minimum of 175 particles were measured but under/over focusing, and the combination of hand and computer sizing could lead to a systematic error in the size estimation. We show the sizes from TEM as more of an estimation. For comparison, SAXS measurements were also carried out, the results of which are shown in Figure 3. The sizes obtained from SAXS suggest somewhat smaller particles, in the range 1.0–1.4 nm, compared to what was seen in TEM, for all liquid substrates. The difficulty in estimating the size distribution of nanoparticles in ionic liquids from SAXS data has been recognized for both Pt^30^ and Ir^31^ particles before. Modeling the ionic liquids as classical organic solvents, without considering their semistructured phase in these types of mixtures, may lead to faulty estimations of nanoparticle sizes. SAXS sizes are included here mainly to support the TEM sizes, as they show particles of the same size order. For further discussion on the SAXS models used in this work, see Supporting Information.

The temperature measurements during each sputter cycle are also presented in Figure 2. During the sputtering process, the plasma heats up the liquid substrate significantly. For each liquid substrate, this temperature increase slows down considerably toward the end of the 5 min sputtering cycle. The highest temperature reached was 198 °C, at the end of the third cycle of sputtering into PEG 600.

We see that the temperature during the sputtering is high, surpassing 100 °C already after 60 s of sputtering and reaching, on average, between 160 and 185 °C after 5 min. In fact, the majority sputtering is performed at temperatures above 140 °C. This should, in case temperature plays an equally important role in Pt nanoparticle size as it has been shown for Au nanoparticles,^3−5^ result in considerably larger nanoparticles compared to the ones found here.

### Influence of Power and Time on Size

**Figure 4:**
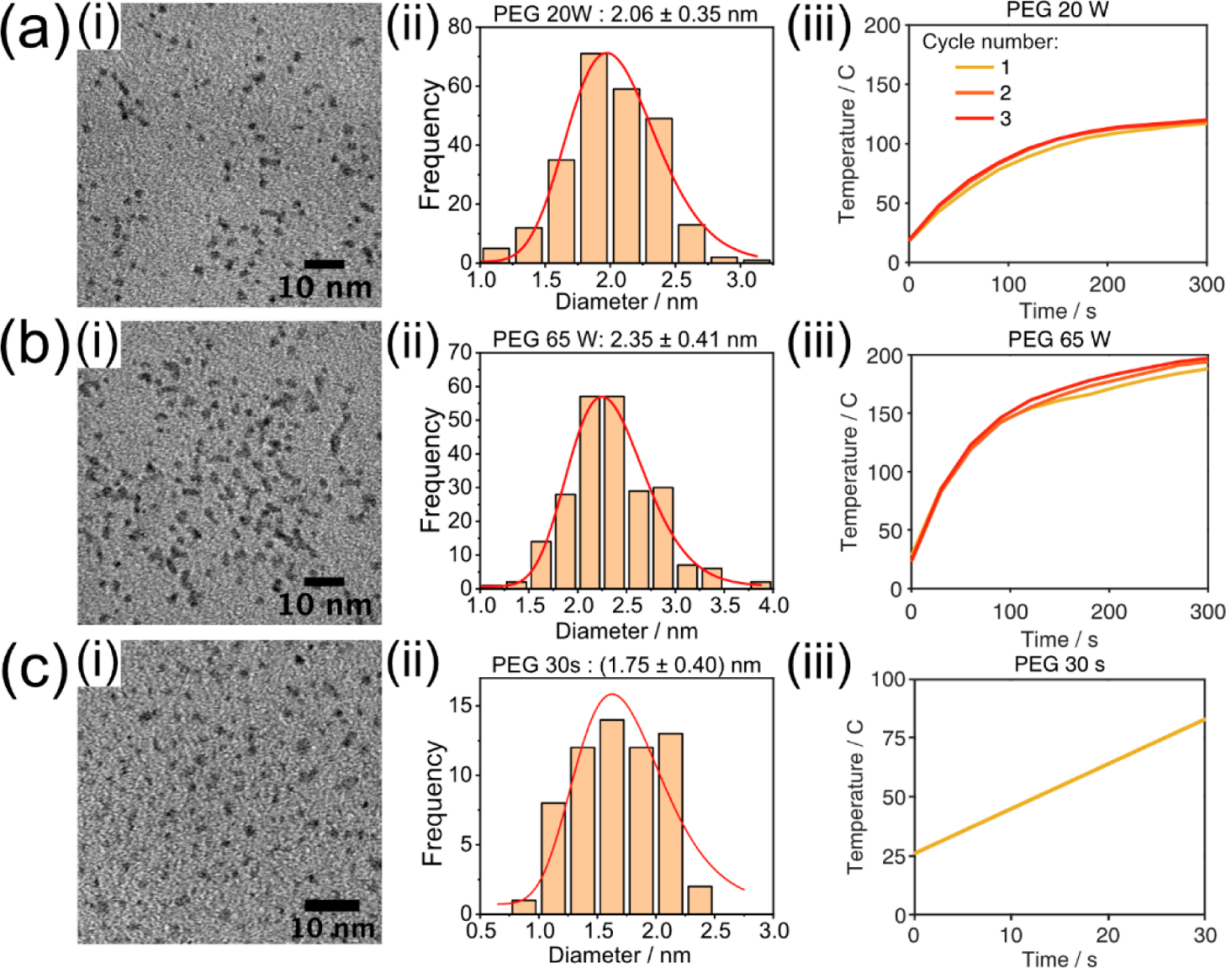
Effect of sputtering power and time on Pt nanoparticle size in PEG. (a) 20 W for 3 × 300 s, (b) 65 W for 3 × 300 s, and (c) just 30 s of sputtering at 50 W. (i) Corresponding TEM images, (ii) nanoparticle size distribution, and (iii) temperature development of the substrate liquid throughout the sputtering cycles.

Varying the power during sputtering changes the Pt nanoparticle size slightly, as shown in Figure 4a,b. Reducing the power by more than half, from 50 to 20 W in Figure 4a, lowers the mean size from 2.2 to 2.1 nm. Increasing the power from 50 to 65 W, in Figure 4b, results in a mean size of 2.4 nm. From Figures 2a(iv) and 4a(iii),b(iii), it is evident that decreasing the sputtering power from 50 to 20 W leads to considerably lower temperatures in the liquid substrate, while increasing the sputtering power to 65 W has little effect on the substrate temperature compared to 50 W. The increase in size that we see for 65 W compared to 50 W we therefore attribute only to the increase in sputtering power, while the size reduction when going from 50 to 20 W might be caused by a combination of decreased power and substrate temperature.

Sputtering for a shorter time produces smaller particles, as shown in Figure 4c. The Pt nanoparticles were, on average, 0.43 nm smaller for just 30 s of sputtering compared to 3 × 300 s. After 30 s, the temperature was around 85 °C, as indicated in Figure 4c(iii). Compared to Au nanoparticles in PEG, this size difference is not much. Hatakeyama’s^4^ experiments show that Au nanoparticles were around 5 nm (2.5 times) larger at 60 °C than at room temperature. We reach 85 °C in 30 s and only see particles smaller (than at 200 °C) by < 0.5 nm. Therefore, with a temperature increase from 85 to 200 °C, there is only a 25% increase in size. Therefore, it appears as temperature does not affect the size of Pt nanoparticles in the same way as for Au nanoparticles.

### Postsputtering Heat Treatment

**Figure 5:**
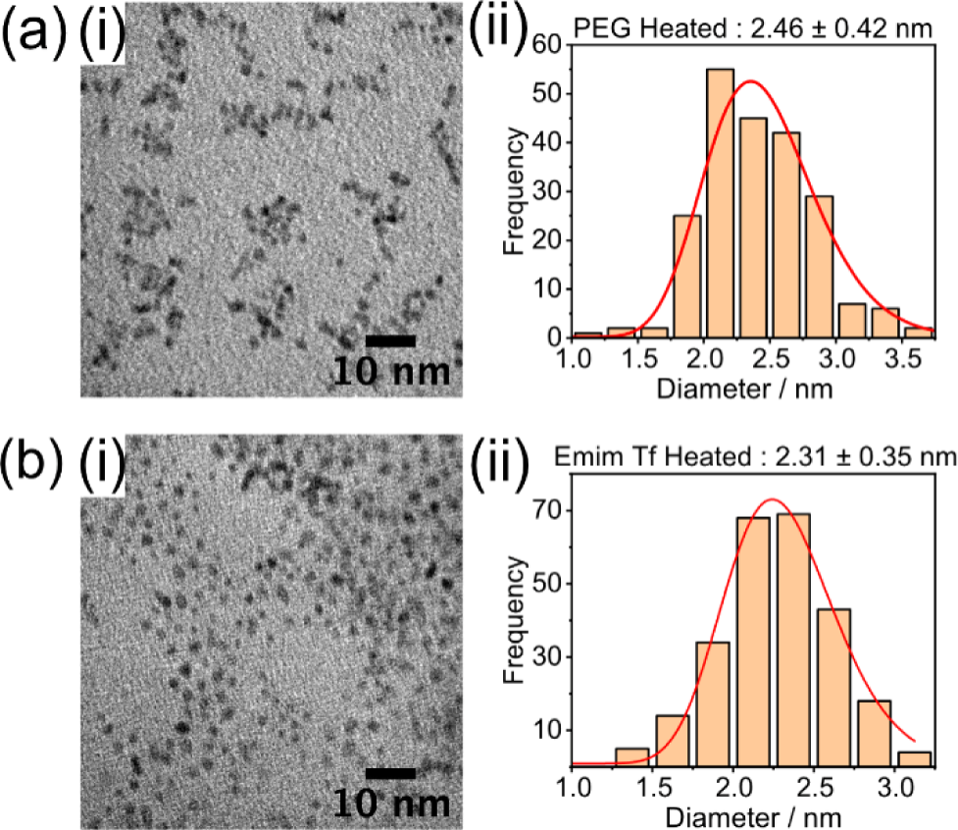
Effect of post sputtering heat treatment on the size of Pt nanoparticles produced in (a) PEG and (b) Emim Tf. Typical TEM images are shown in (i), and the resultant size distributions are shown in (ii).

Figure 5 shows the effect of post heat treatment on particle size. Postsputtering heat treatment to 160 °C for 4 h not only increased particle size slightly but also led to agglomeration, especially pronounced for particles in PEG. While heat treatment does produce larger particles, it also causes agglomeration, so it is therefore not the best method for increasing particle size.

**Figure 6:**
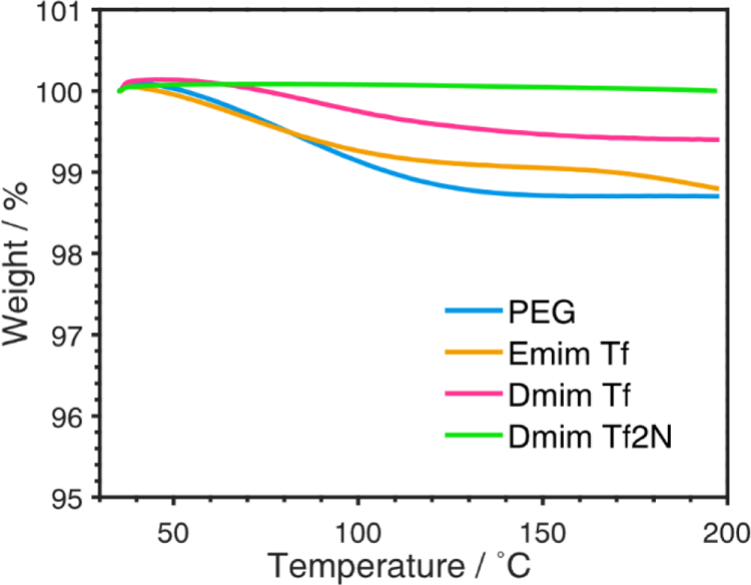
TGA for the liquids used, all loose water during heating but can withstand 200 °C in ambient pressure.

To check the liquid substrate stability at high temperatures, TGA was performed. Figure 6 contains the results of the three pure ionic liquids and PEG 600 heated to 200 °C. All liquid substrates used in this study show high thermal stability at ambient pressure. The loss of mass, which lies within 0–1.3%, depending on the substrate, is mainly attributed to residual water and other impurities contained within the liquids.

During sputtering, the pressure is low, which could lead to different behaviors compared to ambient pressure TGA measurements. However, complete recovery of the 200 μL liquid was possible after sputter deposition, so we conclude that all liquids can withstand these temperatures also during the sputtering process.

### Mechanisms Influencing Particle Size

There does not seem to be a significant difference in the particle size produced in each liquid substrate, with all particles being around 2 nm in diameter. Considering the large temperature change in the liquid during sputtering, this is quite surprising especially compared to the literature on Au nanoparticles. Hatakeyama^7^ found that temperature is very important for particle size, as different liquid substrates have different properties with respect to temperature. They found a significant difference in temperature–size relationship of Au nanoparticles sputtered in PEG and ionic liquids. At 50 °C, Au nanoparticles were nearly 4 nm in Emim Tf and nearly 6 nm in PEG. For a temperature change from 20 to 50 °C, the diameter increased by a factor of 2 in both Emim Tf^7^ (by ∼ 2.5 nm) and in PEG^4^ (by ∼ 4 nm). This would imply that even larger particles and an even larger difference in size would be measured at higher temperatures. We do not see much of a difference in the size of Pt nanoparticles produced in PEG and Emim Tf even at very elevated temperatures (150–200 °C). Compared to Deng’s Pt nanoparticles in PEG,^11^ ours are only 1 nm larger in diameter; however, the temperature of PEG in Deng’s work is unknown. Regardless, we produce Pt nanoparticles around 2 nm which are significantly smaller than previously reported Au nanoparticles and are of a similar size compared to previously reported Pt nanoparticles in PEG, irrespective of the high temperatures. This shows that there is quite a large difference in the temperature-related growth of Pt nanoparticles and Au nanoparticles formed by sputtering in liquid substrates. Similarly, a study by Deng^20^ showed that temperature affected the size of pure Cu nanoparticles but that the effect was minimal when the Cu was alloyed with Pt.

It is well known that Au and Pt behave differently when sputtered onto flat surfaces. Au tends to form large islands and grains, whereas Pt produces smaller grains.^32^ This is in effect what we see also for Pt-sputtered into liquids. It could also be that the Au cluster size in the gas is larger than for Pt. Nevertheless, liquid heating during sputtering is likely positive when making Pt catalyst nanoparticles, as useful sizes are typically above 2 nm in diameter.

The size of incoming metal clusters/atoms has not been measured but likely affects the resulting nanoparticle size greatly. The sputtered material reaches the liquid surface in clusters. It could be considered that the clusters grow inside the liquid due its temperature, and the size achieved is defined by the liquid substrate composition and characteristics. In fact, it seems that Pt cluster size^33^ is different to Au cluster size,^34^ leading to much smaller particles in general. Deng^11^ sputtered directly onto a TEM grid which effectively allowed for cluster size measurement of Pt with 1 s of sputtering. They showed that larger current gave larger Pt nanoparticles, which had the same size as the clusters which came directly from the sputter target. The PEG acted as a support instead of a growth medium. It would be important to separate the effect of gas-phase cluster size on the resultant nanoparticle size in the liquid phase, in order to get a complete picture of the growth of nanoparticles produced by sputtering into liquids.

Temperature also influences the structural organization of imidazolium ionic liquids, with liquids showing greater isotropy with increasing temperature.^35^ Increasing isotropy has been linked to larger particle size. We used PEG 600 for this study, which is also isotropic and produces the largest particles. However, we measure substantial temperature increases in all liquids, and thus, we are not able to reliably elucidate ionic and structural liquid behavior on particle size.

An additional issue with working with such small nanoparticles is their size evaluation, especially nanoparticles that have been produced in ionic liquids. Even with extensive washing of the TEM grid with acetonitrile, the nanoparticles were hard to focus on, likely due to some ionic liquid remaining on the particle surface. We have evaluated TEM images, both standard resolution and high resolution, for particle size; however, there is a significant error attributed to the measurement coming from human-made measurement and computer analysis. Nanoparticles can appear larger or smaller depending on the focus and surface-adsorbed liquid. To further confirm our size analysis, we used SAXS measurements; however, this technique also comes with uncertainty due to the models used to fit and the quality of the sample. Our SAXS fits show the particles to be around 1.0–1.4 nm in diameter for each liquid.

From this study, it is clear that PEG 600 gives the largest particle size. Also, the majority of the PEG is easy to remove from the sample; therefore, this liquid substrate provides a good environment for the production of nanoparticles. To produce particles of a required size, sputtering could be performed at a specific temperature.

**Figure 7:**
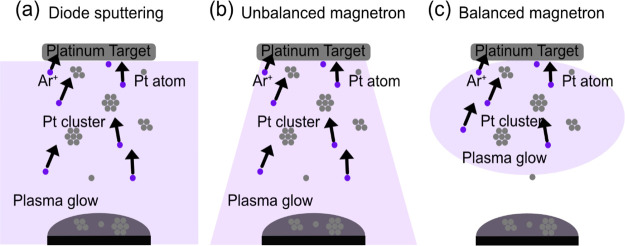
Schematics of different plasma sputtering techniques, highlighting the distribution of plasma in the sputtering chamber during (a) diode, (b) unbalanced magnetron, and (c) balanced magnetron sputtering.

It is slightly tricky to compare particle sizes produced in the same liquids in the literature because there are a few different sputtering methods that exist, each with different parameters. Figure 7 shows a schematic of different sputtering methods. Magnetron sputtering (Figure 7b,c) uses a magnetic field to confine the plasma, so it is less likely to touch the sample surface (depending on the sample working distance), and this allows a fast rate of deposition. In contrast, diode sputtering (Figure 7a) requires a high voltage and high working pressure to maintain a discharge. The plasma is not confined and extends to the substrate and most of the vacuum chamber, heating the entire surroundings. Magnetron sputtering, on the other hand, uses a magnetic field to confine the plasma and enhance the ionization of the sputtering gas (Figure 7c). This allows a lower working pressure and voltage, while the current can be increased significantly to increase the deposition rate. In the future, authors should specify which type of sputtering they are using. For the work presented here, our magnetic field is slightly unbalanced, which causes the plasma to protrude downward onto the sample surface, as shown in Figure 7b, heating it up significantly. While this is not useful if wanting to study small differences between particle sizes in different liquids, the heating effect did help to produce larger particles which were not significantly larger than those produced by others.^11,20,21^ However, more control of the temperature with a heating stage would be preferable for a more constant temperature.

## Conclusions

We have shown that liquid temperature affects the size of Pt nanoparticles produced by sputtering into liquid substrates. However, the effect on particle size is less pronounced than for Au, shown in the literature. This implies that the liquid substrate interaction with the sputtered material is much less important than the sputtering parameters. Size changes after heat treatment show that temperature can be used even after particle formation. Even with heating to over 150 °C, the particle sizes are small, perhaps slightly too small to be used in most applications, such as fuel cells and other catalysts. The next step would be to control the heating during sputtering to have higher liquid temperatures throughout, instead of having the plasma warm the liquid from room temperature. In addition, it would be useful to try other sputtering techniques that can increase cluster size in the gas phase.

## Supporting Information Available

Average temperature evolution during sputtering cycles and SAXS fits for the four different liquid substrates, SAXS fitting procedures, and fitting parameters used (PDF)
