# Supplementary material for: Plasma-Induced Heating Effects on Platinum Nanoparticle Size During Sputter Deposition Synthesis in Polymer and Ionic Liquid Substrates
Source: Langmuir. 2021 Jul 13;37(29):8821–8. doi: 10.1021/acs.langmuir.1c01190 (PMC8397345; doi:10.1021/acs.langmuir.1c01190)
Supplement: Supplementary file 1 — la1c01190_si_001.pdf [file la1c01190_si_001.pdf]

Plasma induced heating effects on platinum nanoparticle size during sputter deposition synthesis in polymer and ionic liquid substrates

Rosemary Brown[1†], Björn Lönn[1], Robin Pfeiffer[1], Henrik Frederiksen[2], Björn Wickman[1*]*

AUTHOR ADDRESS

1. Chemical Physics, Department of Physics, Chalmers University of Technology, 412 96 Gothenburg, Sweden. 2. MC2, Department of Microtechnology and Nanoscience, MC2, Chalmers University of Technology, 412 96 Gothenburg, Sweden.

Temperature Measurements in Liquid Substrates

The various liquid substrates show similar temperature increases due to plasma deposition. These temperature measurements are shown in the main manuscript but here they are presented for an easy comparison between all liquids.

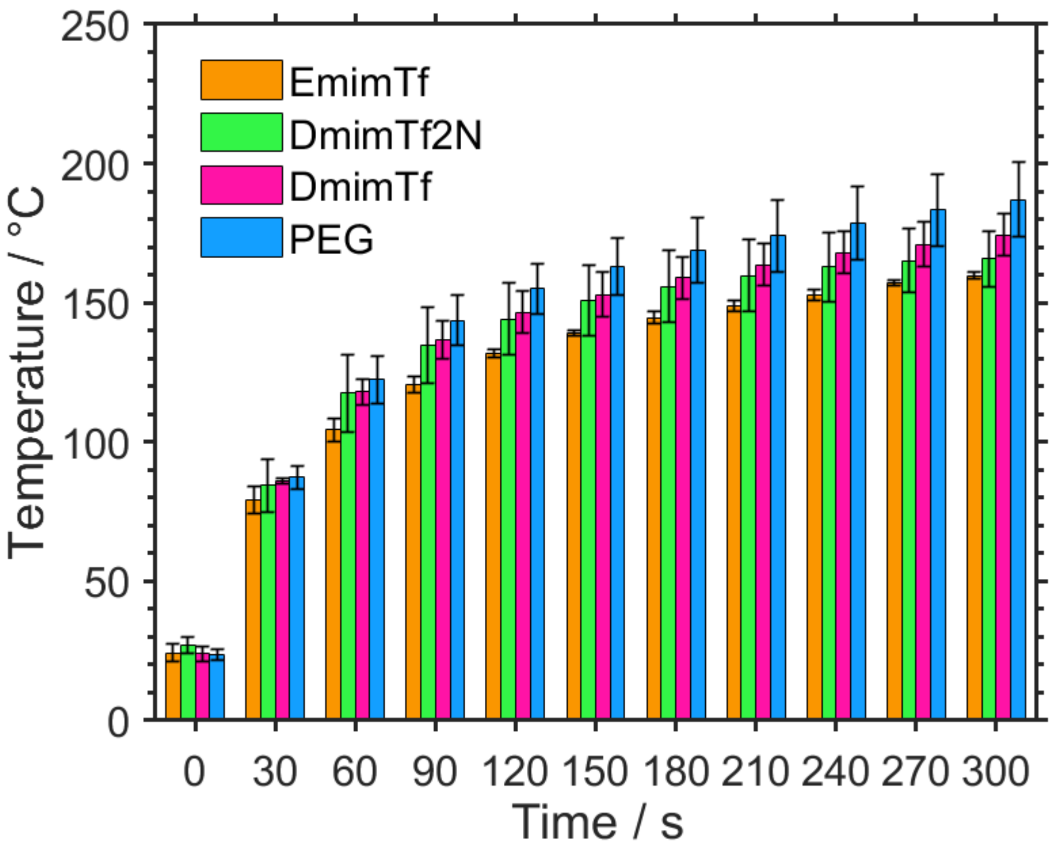

Figure S1. Average temperature measured during sputtering for each liquid. The average is taken from the three sputter cycles of 300s each, followed by 20 minutes rest after each cycle.

SAXS data and fits.

The mean sizes obtained from SAXS fits (performed in Sasview 5.0.3) are smaller than observed in TEM for all liquid substrates. The fits obtained are shown in figure S2. It should be noted that the modelling is based on simply metallic spheres diluted in classical solvents, following the model developed by Guinier [1]. As pointed out by Scheeren et al. [2] and Fonseca et al. [3], in systems like these (ionic liquids + metallic nanoparticles), the ionic liquids constitute a semi-ordered phase, in which the particles are dispersed, complicating the modelling. This has importance for accurate size estimation of transition metal nanoparticles in ionic liquids.

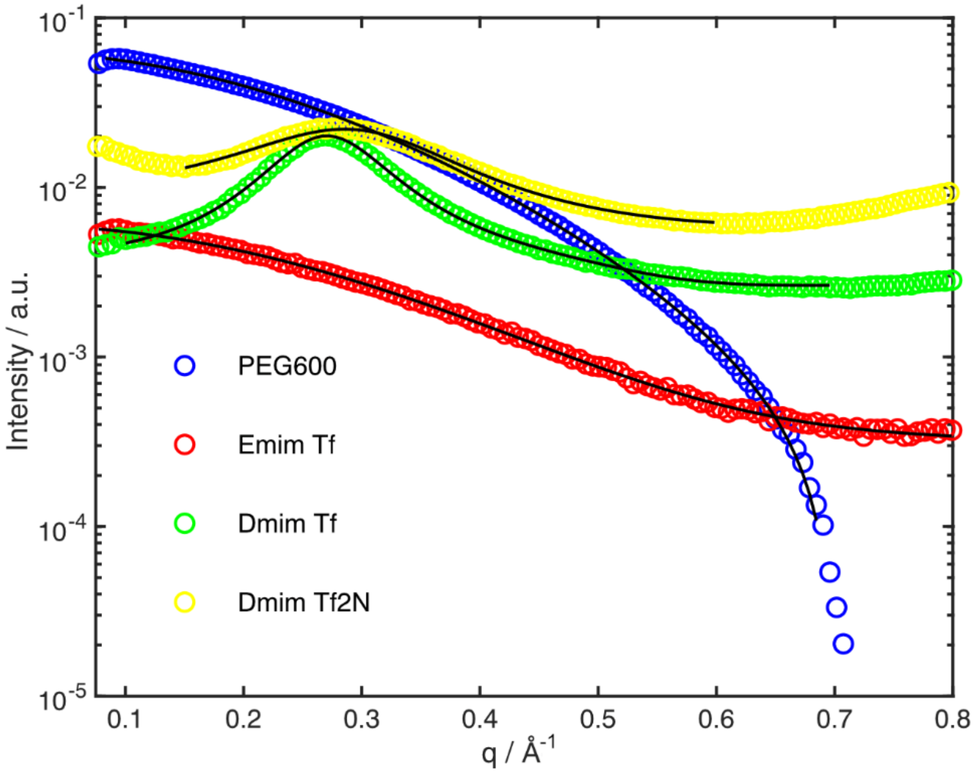

Figure S2. Fits of experimental SAXS data for all liquid substrates. Experimental data shown as rings, with respective fit indicated by solid black lines.

When a structure factor (hayter_msa - Hayter-Penfold Rescaled Mean Spherical Approximation) [4,5] was used in the model (i.e. for the Dmim Tf and Dmim Tf2N liquids), mean sizes showed better agreement with TEM observations. However, this structure factor is made for modelling systems with charged spherical particles in a dielectric solution, which results in charge stabilization due to repulsive forces between the charged spheres. In our liquids, the stabilization of particles is due to steric hinderance by the cation side chains. Dmim-based liquids have the longest side chains and show the need for a structure factor. It is however uncertain how well the charged sphere model can be applied to steric stabilization. As can be seen in table S1, there are large errors connected to some of the involved model parameters when using the hayter_msa structure factor.

Therefore, the mean sizes obtained from SAXS fits, for Dmim Tf and Dmim Tf2N, should be considered with some care. We include it merely as a support for our TEM observations.

**Fitting Parameter**	**PEG 600**<br><br>**Value (Error)**	**Emim Tf**<br><br>**Value (Error)**	**Dmim Tf**<br><br>**Value (Error)**	**Dmim Tf2N**<br><br>**Value (Error)**	**Unit**
Scale	3.4959e-05 (4.9546e-08)	3.7892e-06 (3.838e-08)	0.00014636 (3.8913e-06)	0.00018616 (3.5493e-05)	
Background	-0.00085174 (7.7427e-06)	0.00029834 (5.23e-06)	0.0025286 (2.6273e-05)	0.0058549 (0.00020272)	cm^{-1}
**Sphere**					
SLD (Particles)	137	137	137	137	10^{-6}/Å^2
SLD (Solvent)	10.5	12.2	10.4	11.3	10^{-6}/Å^2
Radius (Median)	4.874 (0.0087237)	4.884 (0.05831)	6.6839 (0.041)	6.4682 (0.36118)	Å
Polydispersity	0.25125 (0.00066785)	0.2255 (0.0047817)	0.12655 (0.017196)	0.12144 (0.049762)	
**Hayter_MSA**					
Volume fraction			0.074401 (0.001402)	0.085945 (0.01133)	
Charge (Particles)			55.808 (12.751)	10.434 (1.21167)	e
Temperature			293	293	K
Salt concentration (Solvent)			9.0085e-08 (0.034954)	1.1166e-10 (0.018846)	M
Dielectric constant (Solvent)			198.68 (38.057)	54.18 (13.665)	

Table S1. Fitting parameters from SAXS fits. Parameters with large errors are highlighted in bold.

Thermocouple placement

A schematic of the thermocouple placement is shown in figure S3.

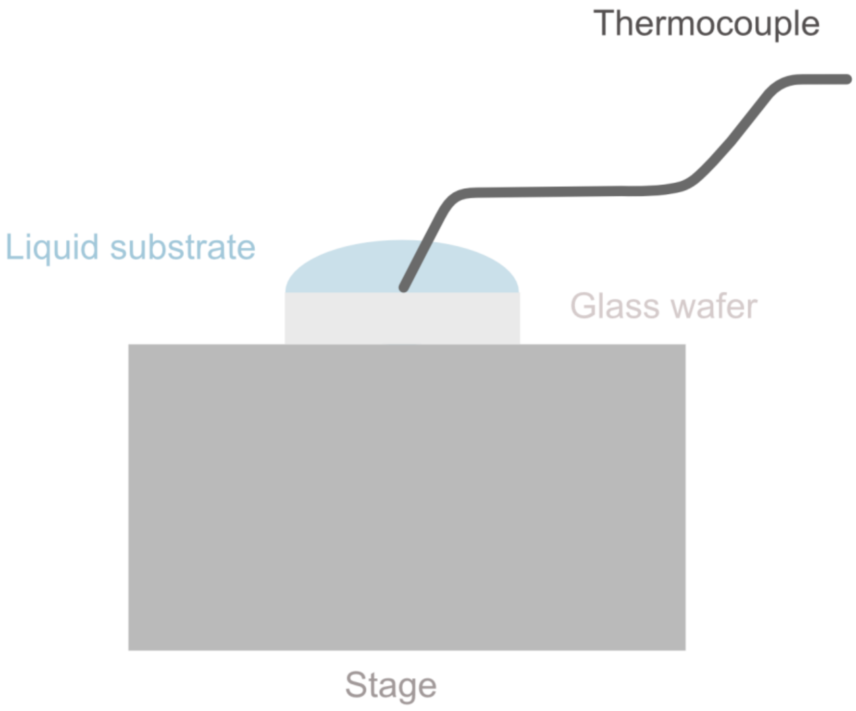

Figure S3. Thermocouple placement in sputtering experiments.

References

(1) Guinier, A.; Fournet, G. *Small-Angle Scattering of X-Rays*; John Wiley & Sons: New York, 1955, 5-65.

(2) Scheeren, C. W.; Machado, G.; Texeira, S. R.; Morais, J.; Domingos, J. B.; Dupont, J. Synthesis and Characterization of Pt(0) Nanoparticles in Imidazolium Ionic Liquids. *J. Phys. Chem. B* **2006**, *110* (26), 13011–13020.

(3) Fonseca, G. S.; Machado, G.; Teixeira, S. R.; Fecher, G. H.; Morais, J.; Alves, M. C. M.; Dupont, J. Synthesis and Characterization of Catalytic Iridium Nanoparticles in Imidazolium Ionic Liquids. *J. Colloid Interface Sci.* **2006**, *301* (1), 193–204.

(4) Hayter, J. B.; Penfold, J. An Analytic Structure Factor for Macroion Solutions. *Mol. Phys.* **1981**, *42* (1), 109–118.

(5) Hansen, J. P.; Hayter, J. B. A Rescaled MSA Structure Factor for Dilute Charged Colloidal Dispersions. *Mol. Phys.* **1982**, *46* (3), 651–656.
